# Status Epilepticus as a Life-Threatening Manifestation of Myxedema Crisis

**DOI:** 10.7759/cureus.21155

**Published:** 2022-01-12

**Authors:** Francisco J Somoza-Cano, Abdul Rahman Al Armashi, Kanchi Patell, Faris Hammad, Keyvan Ravakhah

**Affiliations:** 1 Internal Medicine, St. Vincent Charity Medical Center, Cleveland, USA; 2 Internal Medicine, Northeast Ohio Medical University, Cleveland, USA

**Keywords:** bradycardia, homelessness, myxedema coma, hypothyroidism, status epilepticus, cellulitis, hypothermia

## Abstract

Myxedema coma is a rare life-threatening emergency that usually presents in the elderly during the winter months. The neurological changes caused by uncontrolled hypothyroidism may precipitate seizure activity and its deleterious consequences. A 65-year-old homeless man with a history of non-Hodgkin lymphoma in remission for over 20 years presented to the emergency department (ED) following an episode of syncope. His physical examination was remarkable for hypothermia and bradycardia. Shortly after the admission, he had two tonic-clonic seizures with sphincter relaxation and no recovery between the convulsions. His thyroid-stimulating hormone (TSH) level was 304 uIU/ml. He received appropriate treatment for his condition and was discharged after a full recovery. This case illustrates the catastrophic consequences of long-term uncontrolled hypothyroidism. A high index of clinical suspicion is essential for beginning prompt hormone supplementation in such patients.

## Introduction

Myxedema crisis is defined as severe hypothyroidism that presents with diminished mental status, hypothermia, and symptoms related to the under-functioning of multiple organ systems. It has become an increasingly uncommon event due to the advancements in the treatment of hypothyroidism. However, patients with a complex social history, such as homelessness, are predisposed to this severe manifestation of hypothyroidism. The reduction in oxygen delivery and consumption by the brain, decreased glucose utilization, and reduced cerebral blood flow catalyze an alteration in mental function. Moreover, the inappropriate secretion of antidiuretic hormone in longstanding uncontrolled hypothyroidism can produce hyponatremia and cerebral edema secondary to the expansion of the extracellular fluid volume. These neurological changes may precipitate seizure activity, possibly leading to irreversible hypoxic injury if not promptly recognized [1].

## Case presentation

**Figure 1:**
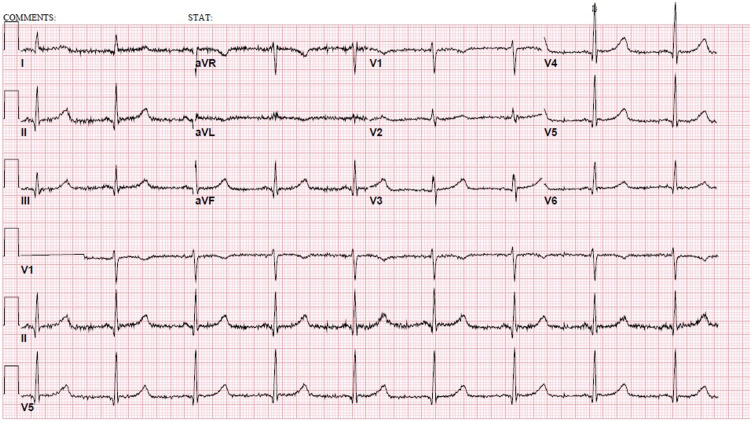
Electrocardiogram A 12-lead EKG was obtained on admission. Sinus bradycardia (52 bpm), a prolonged QTc (503 ms), and shivering artifacts were observed

**Figure 2:**
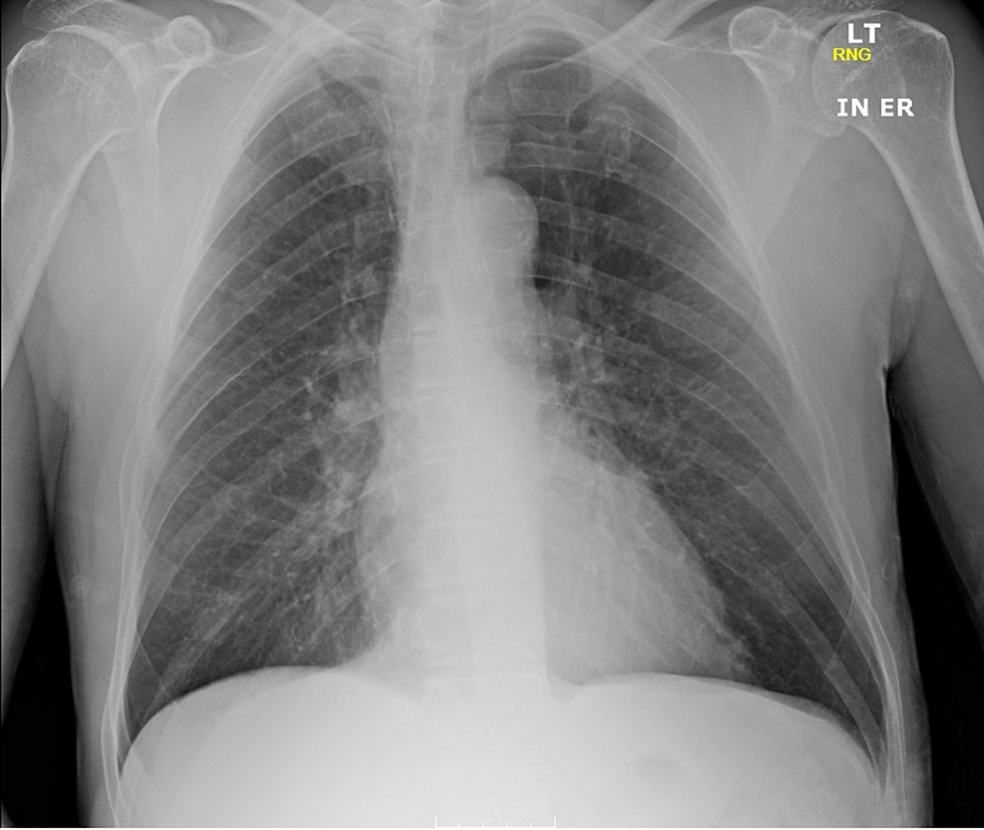
Chest X-ray A single anteroposterior portable chest X-ray was obtained on admission. No acute intrathoracic processes were observed. Mild interstitial prominence in the lungs possibly related to chronic pulmonary disease was documented

A 64-year-old homeless Caucasian man with a past medical history of non-Hodgkin lymphoma in remission since 1996 presented to the emergency department (ED) during the winter following a syncope. Paramedics reported that a bystander had alerted them after a witnessed episode of a sudden loss of consciousness. The patient had no abnormal movements or sphincter relaxation and had a full return to baseline within five minutes. On admission, his blood pressure was 106/57 mmHg, heart rate was 54/minute, respiratory rate was 18/minute, pulse oximetry was 98% on room air, and the temperature was 35.0 °C. The physical examination revealed scaling, edema, erythema, and warmth on his feet that the patient attributed to walking barefoot on the iced pavement. Initial workup showed leukocytosis of 15.9 K/uL (reference range: 3.9-11) but no bandemia. His sodium level was 134 mmol/L (reference range: 135-146) and potassium was 5.9 mmol/L (reference range: 3.5-5.2). Additional electrolytes and kidney function tests were unremarkable. His C-reactive protein was 11.2 mg/L (reference range: 0-3) and the erythrocyte sedimentation rate was 12 mm/h (reference range: 0-20). The EKG showed evidence of sinus bradycardia (52/minute) and prolonged QTc (503 ms) (Figure 1). Chest X-ray (Figure 2) and head CT scan showed no acute processes.

The patient was started on broad-spectrum antibiotics, fluid resuscitation, and passive rewarming; however, shortly afterward, he had two tonic-clonic seizures witnessed by the hospital staff, with bladder relaxation and no recovery between the events. His thyroid-stimulating hormone (TSH) level was found to be 304 uIU/mL (reference range: 0.358-3.74), free thyroxine was 0.34 (reference range: 0.76-1.46), free triiodothyronine was 1.32 pg/mL (reference range: 2.18-3.98), and morning cortisol level was 32.75 ug/dL (reference range: 5.27-22.45). He was transferred to the ICU with worsening bradycardia (41/minute) but normal oxygen saturations on non-invasive ventilation. He was started on intravenous levothyroxine, steroids, and phenytoin. One hour later, he regained consciousness, and his mental status progressively improved. No additional seizure activity was reported. He was discharged after four days of inpatient treatment and made a full recovery thereafter.

## Discussion

Hypothyroidism is a common endocrinological pathology caused by low levels of thyroid hormones. The most common cause of this condition in the United States is Hashimoto's thyroiditis, but low iodine intake remains the most common cause worldwide [2]. In primary hypothyroidism, the thyroid gland is unable to produce adequate amounts of thyroid hormone, while in secondary or central hypothyroidism, the disease lies within the pituitary gland or hypothalamus [2]. Moreover, myxedema crisis is an uncommon life-threatening manifestation of uncontrolled hypothyroidism. Its incidence has been reported to be 0.22 million per year with a mortality rate as high as 60% in certain patient cohorts [2]. Fortunately, it is infrequently encountered in clinical practice currently due to early diagnosis and readily available treatment for hypothyroidism. Moreover, since this entity was first described in 1879, a paucity in scientific research has been observed [1]. This might be secondary to underreporting in conjunction with the progressive decrease in incidence [1,3].

The pathophysiology of myxedema crisis can be usually traced to an event that deranges the body's normal homeostatic state. Patients who have chronic uncontrolled hypothyroidism go through several adaptational changes to preserve normal bodily functions in the absence of normal thyroid hormone levels. These adaptations include chronic peripheral vasoconstriction to prevent hypotension, reduced circulating blood volume to preserve a normal body temperature, and diastolic hypertension [4]. Once a disruptive incident occurs, the body is unable to perpetuate the strenuous effort to maintain homeostasis. Infections, hypothermia, and concomitant diseases are the most common events that trigger this disbalance [4].

Furthermore, the most frequent clinical manifestations are altered mental status, bradycardia, hypotension, hypothermia, and hyponatremia [1,3,4]. Our patient presented with all of the aforementioned clinical manifestations except for hypotension. Moreover, a systematic approach has been suggested for the diagnosis of myxedema crisis based on its three hallmark features: altered mental status, impaired thermoregulation, and a precipitating event [4]. Our patient had unchanged mental status at the time of admission but developed seizure activity shortly afterward. Additionally, he was hypothermic and had feet cellulitis as a precipitating event. Nonetheless, several clinical entities may also produce similar manifestations. Heart failure, hepatic encephalopathy, hypothermia alone, and sepsis may all skew the initial diagnostic approach [1,3,4]. Our patient had an unremarkable chest X-ray without any evidence of pulmonary edema. Additionally, lower extremity edema was only noticed on the feet, where an active infectious process was occurring. Moreover, liver enzymes and kidney function were within normal limits. TSH was more than 60 times its upper limit of normal; unfortunately, no previous records were available for comparison. Even though treatment remains a challenge, our patient had a prompt response after hormone supplementation, antibiotics, anticonvulsants, and supportive measures were started. There is evidence to suggest that thyroxine alone is an effective therapy in myxedema crisis [1].

Finally, the most common neurological presentation of a myxedema crisis does not involve coma but different degrees of obtundation. Seizures and status epilepticus may occur and can be worsened by the concomitant hyponatremia. Our patient initially presented without acute mental status changes. However, the precipitation of the two seizure episodes without recovery between them is diagnostic of status epilepticus [5]. Hyponatremia is seen in up to 50% of patients due to excess vasopressin secretion and/or impaired renal function. Hypothermia progressively aggravates due to the decrease in thermogenesis that ensues after a decrease in the metabolic rate [6]. This same mechanism produces bradycardia and hypotension with prolongation of the QTc, increasing the risk of syncopal events or even torsades de pointes [3,6]. A scoring system has been proposed for the diagnosis of myxedema coma, with a score of more than 60 being potentially diagnostic [7]. Our patient had a score of 70 based on his hypothermia, bradycardia, EKG changes, and hyponatremia. Moreover, after the appropriate treatment was started and the infection was controlled, the patient’s clinical status significantly improved.

## Conclusions

We discussed a case involving an uncommon presentation of uncontrolled hypothyroidism in a 65-year-old male patient. Prompt clinical recognition and appropriate management are paramount for patient survival.
